# Relationship among Serum Progestagens, Cortisol, and Prolactin in Pregnant and Cycling Asian Elephants in Thailand

**DOI:** 10.3390/vetsci9050244

**Published:** 2022-05-22

**Authors:** Patcharapa Towiboon, Kanokporn Saenphet, Chatchai Tayapiwattana, Siriwan Tangyuenyong, Gen Watanabe, Sittidet Mahasawangkul, Janine L. Brown, Chatchote Thitaram

**Affiliations:** 1 Department of Biology, Faculty of Science, Chiang Mai University, Chiang Mai 50200, Thailand; towiboon@gmail.com (P.T.); kanokporn.saenphet@cmu.ac.th (K.S.); 2 Center of Elephant and Wildlife Health, Chiang Mai University, Chiang Mai 50200, Thailand; brownjan@si.edu; 3 Center of Biomolecular Therapy and Diagnosis, Department of Medical Technology, Faculty of Associated Medical Sciences, Chiang Mai University, Chiang Mai 50200, Thailand; chatchai.t@cmu.ac.th; 4 Department of Companion Animals and Wildlife Clinics, Faculty of Veterinary Medicine, Chiang Mai University, Chiang Mai 50100, Thailand; siriwan.tangy@cmu.ac.th; 5 Animal Physiology, Department of Veterinary Medicine, Faculty of Agriculture, Tokyo University of Agriculture and Technology, Tokyo 183-8538, Japan; gen@cc.tuat.ac.jp; 6 Thai Elephant Conservation Center, National Elephant Institute, Forest Industry Organization, Lampang 52190, Thailand; msittidej@hotmail.com; 7 Center for Species Survival, Smithsonian National Zoo Conservation Biology Institute, Front Royal, VA 22630, USA

**Keywords:** progestagens, cortisol, prolactin, pregnancy, ovarian cycle, Asian elephant

## Abstract

The aim of this study was to examine relationships among serum progestagens, cortisol, and prolactin in pregnant and normal cycling Asian elephants living in tourist camps in northern Thailand. Samples were collected twice a month for 22 months from nine elephants. Of those, four were pregnant (24.3 ± 2.9 years of age; range 21–28 years) and five (20.2 ± 9.6 years; range 8–34 years) exhibited normal ovarian cycles based on serum progestagen analyses. Gestation was divided into three periods: 1st (week 1–31), 2nd (week 32–62), and 3rd (week 63 to parturition), while the estrous cycle was divided into the follicular and luteal phases. Serum progestagens were higher during the luteal phase of the cycle (*p* < 0.003), whereas cortisol and prolactin were similar. In pregnant elephants, there were no differences in serum progestagens or cortisol concentrations across the three gestational periods, whereas prolactin concentrations increased significantly during the 2nd and 3rd periods (*p* < 0.0001). By contrast, prolactin concentrations in nonpregnant elephants were consistently low throughout the ovarian cycle. In one cycling female, prolactin concentrations were similar to pregnant elephants, perhaps because she was an allomother to two calves. Another cycling female exhibited consistently elevated cortisol concentrations, 5 to 10 times higher than the other elephants. There were no correlations between serum progestagens, cortisol, and prolactin throughout gestation; however, serum progestagens and cortisol were positively related in cycling elephants (r = 0.386, *p* < 0.001). From our results, there were a number of individual differences in reproductive hormonal patterns, so it is important to develop personalized monitoring programs for each elephant to enhance breeding success and create sustaining captive populations of elephants in Asia.

## 1. Introduction

In Thailand, there are approximately 3100–3600 wild elephants in 69 protected areas, and 3783 captive elephants that are mostly (95%) privately owned [1], with 2700 elephants (70.7%) located in 250 camps that are part of the tourist industry [2]. Males and females are generally kept separated and occasionally brought together for breeding, and overall reproductive rates are low, although it is not known if reproductive failure is physiological or management-related. Therefore, studies of elephant reproduction are warranted, including monitoring hormones to help with estrus detection, pregnancy diagnosis, parturition prediction, and postpartum management.

Pregnancy in elephants can be diagnosed by measuring circulating progestagens, which are continuously elevated until parturition, declining to baseline 3–5 days before birth [3]. Given the length of the normal luteal phase (8–10 weeks), progestagens concentrations should be monitored continuously once or twice a week for at least 4 months to confirm a pregnancy [3,4]. Another useful pregnancy test is to measure prolactin immunoactivity (ir-prolactin). After 4–7 months of gestation, ir-prolactin increases up to 100-fold, peaks at 11–14 months, and remains high until birth in both Asian and African elephants [5,6,7,8]. The source of high ir-prolactin during gestation is primarily placental [8], similar to lactogenic hormones in other mammals [9]. Prolactin and placental lactogens are luteotropic in other species and enhance CL progestagens production [10,11], which is important for elephants because the placenta is steroidogenically inactive (African: [12]). Measurement of serum prolactin past 7 months of gestation is a reliable pregnancy test, even with a single sample, unlike progestagens, which require longitudinal sampling [13]. In both species, significant increases in serum cortisol are observed in the weeks before and again at birth [6,14], suggesting an important role in the initiation of parturition.

During the estrous cycle, Fanson et al. [15] showed that serum progesterone and cortisol concentrations were correlated in Asian elephants, but offset by several weeks. Cortisol concentrations increased during the first half of the follicular phase and were highest during the second half, which was similar to the study of Bechert et al. [16], which found that serum cortisol and progesterone were negatively correlated. For prolactin, there is a notable species difference in secretary patterns throughout the cycle. In Asian elephants, concentrations show no clear patterns, and generally remain at baseline throughout the cycle [17]. By contrast, prolactin concentrations are elevated during the follicular phase, low during the luteal phase in African elephants [18,19,20], and are positively correlated with serum cortisol [16].

To increase reproductive success in captive Asian elephants in Thailand, early pregnancy detection is key, which would allow camp owners to adjust management to reduce the physical intensity of workloads and provide proper nutrition to prevent problems with dystocia or stillbirths. One hormone often used as a proxy for stress is cortisol, a steroid hormone produced by the adrenal cortex that is released in response to both internal and external stressors, including in elephants [21,22]. Long-term elevations in cortisol associated with chronic stress can affect animal health [23,24], body weight, behavior, immune function, and the reproductive system (e.g., acyclicity, irregular cycling, infertility, abortion, stillbirths) [25,26]. Thus, reproductive and stress-hormone monitoring during pregnant and nonpregnant periods in elephants is important and could provide valuable information for improving reproductive management.

Given the limited information on hormonal patterns in elephants living in range countries, this study aimed to longitudinally monitor progestagens, prolactin, and cortisol in pregnant and nonpregnant Asian elephants working in tourist camps in northern Thailand. Hormone concentrations between the follicular and luteal phases of the estrous cycle, and during early, mid, and late pregnancy were compared. In addition, relationships among these hormones throughout these reproductive phases also were calculated. The ultimate goal is to understand reproductive processes in elephants and apply that information to create more successful breeding programs in Thailand.

## 2. Materials and Methods

### 2.1. Animals

Four pregnant (E1–E4) (24.3 ± 2.9 years of age; range, 21–28 years) and five nonpregnant, normal cycling (E5–E9) (20.2 ± 9.6 years; range, 8–34 years) elephants were used in this study (Table 1). All elephants were from three tourist camps in Chiang Mai province that participated in shows or riding activities for tourists. Blood samples were collected twice a month from an ear vein by elephant veterinarians at each facility, kept at room temperature for 1–2 h, and centrifuged at 2000× *g* for 5 min to obtain serum. Serum samples were stored in 1.5 mL aliquots at −20 °C until analysis. Blood samples were collected as part of a routine healthcare program provided by Chiang Mai University.

### 2.2. Hormonal Analysis

Progestagens were analyzed by a single-antibody enzyme immunoassay (EIA) following Thitaram et al. [27]. In brief, the progesterone EIA used a monoclonal antiprogesterone antibody (1:10,000; CL425), horseradish peroxidase (HRP)-conjugated progesterone label (1:40,000; C. Munro, University of California-Davis, Davis, CA, USA), and progesterone standards (catalog #P0130; Sigma Chemical Co., St. Louis, MO, USA). The assay sensitivity was 0.03 ng/mL The cortisol EIA utilized a cortisol polyclonal antibody (R4866 antisera, C. Munro) at a dilution of 1:10,000 and cortisol HRP (C. Munro) at a dilution of 1:15,000.

Prolactin was analyzed following Yamamoto et al. [19] by a double-antibody heterologous radioimmunoassay, using an antihuman prolactin rabbit serum (NIDDK-anti hPRL3), iodinated ovine prolactin (NIDDK-oPRL-I-2), ovine prolactin standard (NIDDK-RP-2), and a goat antirabbit gamma globulin second antibody, validated previously for elephants [5,17,19]. Assay sensitivities were 0.03, 0.17, and 0.25 ng/mL for progesterone, cortisol, and prolactin. The inter-and intra-assay coefficients of variation for all assays were less than 15% and 10%, respectively.

### 2.3. Statistical Analysis

Average serum progestagens, cortisol, and prolactin concentrations of pregnant elephants were compared across three gestational periods: 1st (0–31 weeks); 2nd (32–62 weeks); 3rd (63 weeks to parturition), and in cycling elephants were compared between follicular and luteal phases by General Linear Model repeated-measure analysis (α = 0.05) (SPSS 18.0; SPSS Inc., Chicago, IL, USA). Baseline progestagens were calculated using an iterative approach described in Glaeser et al. [28]. All data points with values above the mean plus 1.5 times the SD were removed, and the process repeated until no values exceeding the mean < 1.5xSD remained. The remaining data points defined the baseline for that individual. Correlations between hormones were analyzed using a Spearman’s correlation test (α = 0.05) (SPSS). Data are presented as mean ± SEM.

## 3. Results

Elephants E1–E4 were pregnant at the beginning of the study (1st to 20th month), while the other females exhibited normal ovarian cycles. One cycling female (E5) had given birth 3 months before the start of the study and was still nursing a calf. She also exhibited lactational anestrus from the 1st to the 18th week of the study before resuming cycling.

**Figure 1:**
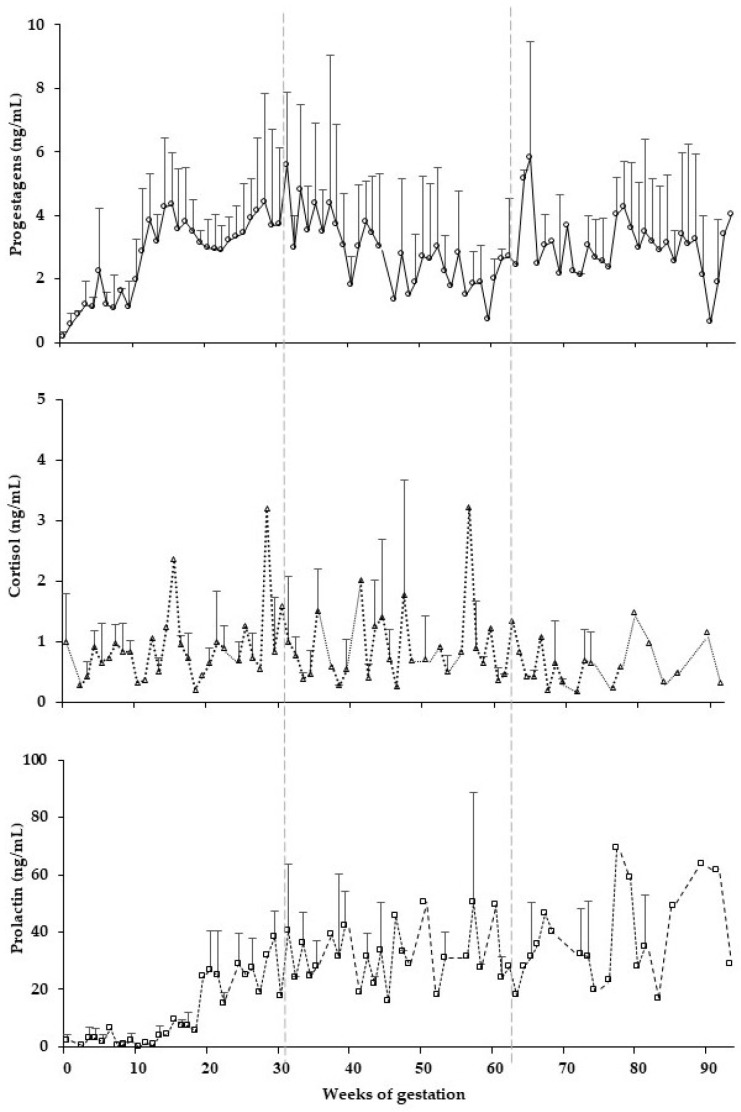
Mean (±SEM) concentrations of serum progestagens, cortisol, and prolactin in four pregnant elephants (E1–E4) over the weeks of gestation.

Serum progestagens concentrations were elevated throughout gestation and similar in concentration to the luteal phase of cycling elephants (*p* < 0.0001). Serum cortisol concentrations fluctuated in all females, but did not differ statistically among gestation or ovarian cycle periods (Table 2). Serum prolactin concentrations were low postconception and then increased at 20 weeks, remained elevated throughout the 2nd and 3rd gestational periods (Figure 1), and were higher than overall means in cycling elephants (*p* < 0.001). Overall serum progestagens and cortisol concentrations were not different between gestational periods, whereas serum prolactin was higher in the 2nd and 3rd periods compared to the 1st (*p* < 0.0001) (Table 2).

**Figure 2:**
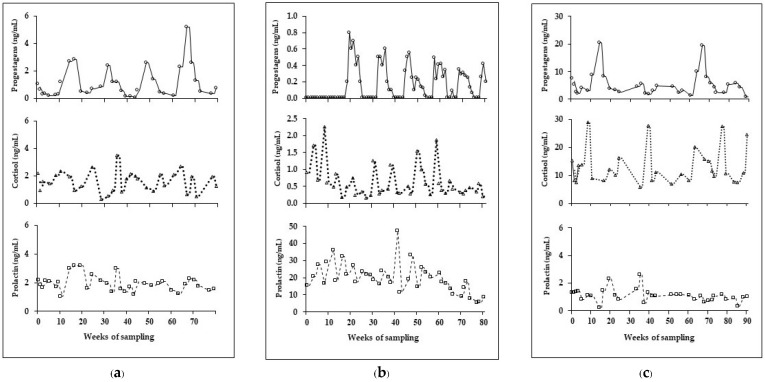
Individual profiles of serum progestagens, cortisol, and prolactin concentrations in (**a**) normal cycling elephant (E9); (**b**) cycling elephant with elevated concentrations of prolactin (E5); and (**c**) cycling elephant with elevated cortisol (E6).

The average estrous cycle length for the five females was 13.0 ± 1.1 weeks (follicular phase, 4.5 ± 0.3 weeks; luteal phase, 14.2 ± 0.9 weeks) (*n* = 17 cycles). In all females, serum progestogens and cortisol were higher during the luteal compared to the follicular phase, whereas there were no differences between cycle phases for serum prolactin (Table 2, Figure 2a). In one female (E5), serum prolactin values were excluded from mean calculations because concentrations were continually elevated and an order of magnitude higher than any other cycling female (Table 3, Figure 2b). Moreover, this elephant had the lowest mean serum progestagens concentrations for both follicular (less than a tenth) and luteal (less than a quarter) phases compared to the other cycling females. Elephant E6 exhibited the highest average concentrations of both serum progestagens and cortisol, which was at least five times higher than the other elephants (Table 3, Figure 2c). Serum cortisol concentrations in pregnant elephants were overall lower than those in cycling elephants (*p* < 0.001). There were no correlations among serum progestagens, cortisol, and prolactin in pregnant elephants. However, there was a positive relationship between progestagens and cortisol in cycling elephants (*p* < 0.001) (Table 4).

## 4. Discussion

In this study, each elephant—both pregnant and normal cycling—exhibited a distinctive pattern, confirming the need for individual reproductive monitoring and screening to assess reproductive status and fertility potential. Prolactin concentrations in pregnant elephants were three-fold higher in the 2nd period of gestation, while those of nonpregnant elephants were low throughout the cycle, similar to previous studies [5,6,7,29,30,31]. Increases in gestational prolactin facilitate mammary gland development, milk production, and maintenance of corpus luteum function, as well as promote progestagens secretion [32,33], and have been proposed to support pregnancy and fetal development in elephants [5,16,34]. In cycling elephants, prolactin concentrations did not differ between follicular and luteal phases, which represents a significant species difference with African elephants, where prolactin exhibits a clear pattern of elevated concentrations during the follicular phase [19,20]. One outlier in this study was a female that was in lactational anestrus at the beginning of the study and then began cycling. This female exhibited consistently elevated prolactin until about the fifth postpartum cycle, at which time concentrations slowly declined to baseline. This female was still nursing a calf and known to be a good allomother to other calves. She was caring for two stepbabies during this study, thus supporting a maternal role for prolactin in elephants. We are not aware of other reports in elephants of elevated prolactin continuing after birth during lactation [5,17,31,34,35], so this female appears to be an anomaly. In addition, the normal postpartum anestrous period generally lasts 12 to 24 months [36], whereas this female resumed cycling after only 18 weeks and while prolactin was still high. Lueders et al. [37] suggested that high concentrations of serum prolactin in cycling elephants might extend the luteal phase, but our data showed this female exhibited regular cycle lengths.

Cortisol concentrations in cycling elephants were higher than those in pregnant females and differed between follicular and luteal phases. This was similar to the study of Oliveira et al. [38], which showed that serum cortisol concentrations in cycling Asian elephants were double those of pregnant animals. Adrenal function depends on many factors and in elephants can be affected by the captive environment, as well as nutrition, health, and tourist activities [39]. Hypercortisolemia is related to overstimulation of the hypothalamic–pituitary–adrenal (HPA) axis, with increased secretion of hypothalamic corticotropin-releasing hormone (CRH) and/or pituitary adrenocorticotropic hormone (ACTH) resulting in elevated glucocorticoid secretions such as cortisol. Glucocorticoids can inhibit gonadotropin-releasing hormone (GnRH), which decreases gonadotropin production, and has been shown to impair ovarian cycles in cattle, rodents, and nonhuman primates [40,41]. However, in E6, high serum cortisol had no effect on estrous cyclicity, as has also been found in previous studies [35,38,42]. There were no significant differences in overall mean serum cortisol concentrations across the three gestational periods, which contrasts with Meyer et al. [6]. That study reported two distinct peaks in late gestation in both African and Asian elephants, which were evident because of the nearly daily sampling regimen used in the last month or two. In cycling elephants, cortisol was significantly higher in the luteal phase, which contrasts with previous studies that reported higher cortisol during the follicular phase [15], or no differences between follicular and luteal phases [35,38], which again might be due to different sampling frequencies.

There were no significant correlations between serum cortisol and progestagens, or cortisol and prolactin in pregnant elephants, although the latter approached significance similar to that reported for African elephants [16]. In our study, serum cortisol and progestagens were correlated in cycling elephants due to higher concentrations during the luteal phase. Our data contrast with that of Fanson et al. [15], which found higher serum cortisol during the follicular phase in Asian elephants, and with Bechert et al. [16], who found a negative correlation with serum progestagens, suggesting there may be slight species differences.

## 5. Conclusions

There were no significant relationships between serum progestagens, cortisol, and prolactin in pregnant elephants, whereas serum progestagens and cortisol were positively correlated in cycling females. Serum prolactin concentrations were high throughout gestation, significantly so in the 2nd and 3rd periods. Surprisingly, in one female, high serum prolactin concentrations were found throughout the postpartum period, even after she resumed cycling while lactating. This finding matches the observation that she is a particularly good allomother and supports prolactin being a maternal hormone in elephants. Perhaps measures of prolactin could be used to identify potential allomother candidates, especially to help first-time mothers that have had little exposure to calves. We also found that high cortisol in one female did not disrupt normal ovarian cycle activity. Thus, it is important to take into consideration individual differences in hormone patterns throughout different reproductive states in the assessment of reproductive potential.

## Figures and Tables

**Table 1:** Summary of the demographics, reproductive status, work activities, and body condition scores of elephants evaluated in the study.

Elephant	Age	Sampling Date	Parturition Date	Camp	Work Type	Reproductive State	Parity	BCS
E1	24	June 2005 to March 2007	7 March 2007	A	Saddle riding	Pregnant	2	4
E2	24	August 2007 to May 2009	31 May 2009	A	Saddle riding	Pregnant	2	4.5
E3	21	December 2003 to Sep 2005	30 Sep 2005	B	Saddle riding	Pregnant	1	3.5
E4	28	June 2004 to August 2005	14 March 2006	B	Saddle riding	Pregnant	2	3.5
E5	23	August 2004 to March 2006	6 May 2004	A	Saddle riding	Postpartum and cycling	1	4.5
E6	20	November 2014 to August 2016	-	C	Bareback riding	Cycling	2	4
E7	16	November 2014 to August 2016	-	C	Bareback riding	Cycling	Nulliparous	3
E8	8	November 2014 to August 2016	-	C	Bareback riding	Cycling	No data	4
E9	34	November 2014 to August 2016	-	C	Bareback riding	Cycling	Nulliparous	4.5

**Table 2:** Mean (±SEM) serum progestagens, cortisol, and prolactin concentrations (ng/mL) across different phases of the estrous cycle and period of pregnancy.

Status	Period	Progestagens (ng/mL)	Cortisol (ng/mL)	Prolactin (ng/mL)
Pregnant	1st	2.74 ± 0.24 ^a^	0.76 ± 0.08 ^a^	8.59 ± 1.76 ^a^
	2nd	3.40 ± 0.22 ^a^	0.91 ± 0.11 ^a^	31.15 ± 2.53 ^b^
	3rd	3.09 ± 0.21 ^a^	0.62 ± 0.08 ^a^	36.47 ± 3.34 ^b^
	Overall mean	2.92 ± 0.11	0.81 ± 0.06	24.82 ± 1.64
Cycling	Follicular phase	0.23 ± 0.05 ^a^	2.80 ± 0.77 ^a^	2.06 ± 0.15 ^a,^*
	Luteal phase	2.54 ± 0.29 ^b^	6.52 ± 1.00 ^b^	1.54 ± 0.24 ^a,^*
	Overall mean	1.68 ± 0.19	3.65 ± 0.44	1.86 ± 0.08 *

* Excludes E5 that had abnormally high prolactin concentrations. Mean values in the same row bearing different superscript letters (a, b) were significantly different (*p* < 0.05).

**Table 3:** Individual mean (±SEM) progestagens, cortisol, and prolactin concentrations in pregnant and cycling elephants.

Status	Elephant	Progestagens (ng/mL)		Cortisol (ng/mL)	Prolactin (ng/mL)
Pregnant	E1	3.06 ± 0.21 ^b^		1.08 ± 0.13 ^b^	16.61 ± 2.77 ^a^
	E2	3.81 ± 0.30 ^c^		0.54 ± 0.06 ^a^	20.07 ± 3.15 ^a^
	E3	2.86 ± 0.22 ^b^		0.73 ± 0.13 ^ab^	27.64 ± 3.84 ^b^
	E4	1.53 ± 0.09 ^a^		1.05 ± 0.14 ^b^	18.41 ± 2.81 ^a^
Cycling		Follicular phase	Luteal phase		
	E5	0.01 ± 0.01 ^a^	0.39 ± 0.04 ^a^	0.63 ± 0.09 ^a^	20.95 ± 1.42 ^d^
	E6	1.91 ± 0.16 ^c^	6.39 ± 1.15 ^c^	13.82 ± 1.32 ^c^	1.09 ± 0.08 ^a^
	E7	0.39 ± 0.02 ^b^	1.41 ± 0.22 ^b^	1.66 ± 0.17 ^b^	2.90 ± 0.16 ^c^
	E8	0.34 ± 0.06 ^b^	3.11 ± 0.56 ^bc^	2.07 ± 0.28 ^b^	1.47 ± 0.11 ^a^
	E9	0.16 ± 0.03 ^ab^	1.09 ± 0.19 ^b^	1.61 ± 0.15 ^b^	1.93 ± 0.09 ^b^

^a,b,c,d^ Column values across individual elephants are significantly different (*p* < 0.05).

**Table 4:** Correlations between hormone concentrations in pregnant and cycling elephants.

Hormone	Pregnant		Cycling	
	r	*p*	r	*p*
Progestagens and cortisol	−0.114	0.224	0.386	0.000
Cortisol and prolactin *	−0.092	0.330	−0.030	0.781
Progestagens and prolactin *	0.177	0.058	−0.046	0.593

* Excludes E5 that had abnormally high prolactin concentrations.

## Data Availability

Not applicable.
